# Model-estimated relationship between elementary school-related SARS-CoV-2 transmission, mitigation interventions, and vaccination coverage across community incidence levels

**DOI:** 10.1101/2021.08.04.21261576

**Published:** 2021-11-16

**Authors:** John Giardina, Alyssa Bilinski, Meagan C. Fitzpatrick, Emily A. Kendall, Benjamin P. Linas, Joshua Salomon, Andrea L. Ciaranello

**Affiliations:** Center for Health Decision Science, Harvard T.H. Chan School of Public Health, Boston, MA; Department of Health Services, Policy, and Practice and Department of Biostatistics, Brown School of Public Health, Providence, RI, USA; Center for Vaccine Development and Global Health, University of Maryland School of Medicine, Baltimore, MD; Division of Infectious Diseases, Johns Hopkins University School of Medicine, Baltimore, MD; Boston University Schools of Medicine and Public Health, Boston Medical Center, Boston, MA; Center for Health Policy and Center for Primary Care and Outcomes Research, Stanford University School of Medicine, Stanford, CA; Division of Infectious Disease and Medical Practice Evaluation Center, Massachusetts General Hospital, Boston, MA, USA

## Abstract

**Background:**

While CDC guidance for K-12 schools recommends indoor masking regardless of vaccination status, final decisions about masking in schools will be made at the local and state level. The impact of the removal of mask restrictions, however, on COVID-19 outcomes for elementary students, educators/staff, and their households is not well known.

**Methods:**

We used a previously published agent-based dynamic transmission model of SARS-CoV-2 in K-12 schools to simulate an elementary school with 638 students across 12 scenarios: combinations of three viral infectiousness levels (reflecting wild-type virus, alpha variant, and delta variant) and four student vaccination levels (0%, 25%, 50% and 70% coverage). For each scenario, we varied observed community COVID-19 incidence (0 to 50 cases/100,000 people/day) and mitigation effectiveness (0–100% reduction to in-school secondary attack rate), and evaluated two outcomes over a 30 day period: (1) the probability of at least one in-school transmission, and (2) average increase in total infections among students, educators/staff, and their household members associated with moving from more to less intensive mitigation measures.

**Results:**

Over 30 days in the simulated elementary school, the probability of at least one in-school SARS-CoV-2 transmission and the number of estimated additional infections in the immediate school community associated with changes in mitigation measures varied widely. In one scenario with the delta variant and no student vaccination, assuming that baseline mitigation measures of simple ventilation and handwashing reduce the secondary attack rate by 40%, if decision-makers seek to keep the monthly probability of an in-school transmission below 50%, additional mitigation (e.g., masking) would need to be added at a community incidence of approximately 2/100,000/day. Once students are vaccinated, thresholds shift substantially higher.

**Limitations:**

The interpretation of model results should be limited by the uncertainty in many of the parameters, including the effectiveness of individual mitigation interventions and vaccine efficacy against the delta variant, and the limited scope of the model beyond the school community. Additionally, the assumed case detection rate (33% of cases detected) may be too high in areas with decreased testing capacity.

**Conclusion:**

Despite the assumption of high adult vaccination, the risks of both in-school SARS-CoV-2 transmission and resulting infections among students, educators/staff, and their household members remain high when the delta variant predominates and students are unvaccinated. Mitigation measures or vaccinations for students can substantially reduce these risks. These findings underscore the potential role for responsive plans, where mitigation is deployed based on local COVID-19 incidence and vaccine uptake.

## INTRODUCTION

CDC recommends in-person education for all K-12 students, with COVID-19 mitigation measures including distancing, ventilation, and indoor masking regardless of vaccination status.^1^ Vaccination is now authorized for children <12. In communities with high vaccination rates and low COVID-19 incidence, or where masking is less widely accepted, schools may remove masks and other mitigation requirements, with uncertain impact on COVID-19 outcomes for elementary students, educators/staff, and their households.

## METHODS

We used an agent-based dynamic transmission model of SARS-CoV-2 in schools. Model structure and data inputs are described in previous publications; the Supplement describes parameterization specific to this analysis.^2^ Current and prior^2^ reporting adhere to CHEERS guidelines; this was designated not human subjects research.

We simulated an elementary school (30 separate classes, 638 students, 60 educators/staff) across 12 different combinations of: three viral infectiousness levels (reflecting wild-type virus, alpha variant, and delta variant) and four student vaccination levels (0%, 25%, 50%, and 70% coverage). We assumed that 70% of adults (educators/staff and adult household members of students and educators/staff) were vaccinated (sensitivity analyses for 50% adult vaccination coverage can be found in the Supplement). For each scenario, we varied observed community COVID-19 incidence (0–50 cases/100,000 people/day, 33% of cases detected) and mitigation effectiveness (0–100% reduction to in-school secondary attack rate). Without clinical data for individual mitigation measure effectiveness, we created examples based on observational data, particle and aerosol studies, and expert opinion, to reflect three levels of in-school mitigation intensiveness: A) ventilation and handwashing only (20–40% effectiveness); B) masking plus ventilation/handwashing (70%−80%); and C) combined masking, distancing, cohorting, handwashing, and ventilation (90–100%). These ranges are highly uncertain (Supplement).

We evaluated two primary outcomes over a 30-day period: 1) probability of any in-school SARS-CoV-2 transmission with varying mitigation effectiveness, and 2) average increase in total infections among students, educators/staff, and their household members (“immediate school community”) associated with moving from more to less intensive mitigation measures (e.g., unmasking). We projected the anticipated increased in cases associated with three discrete changes in mitigation effectiveness, reflecting the midpoints or bounds of the A and B mitigation scenarios above: 70% to 40% mitigation effectiveness (smaller decrease); 75% to 35% effectiveness (moderate decrease); and 80% to 20% effectiveness (larger decrease).

## RESULTS

**Figure 1:**
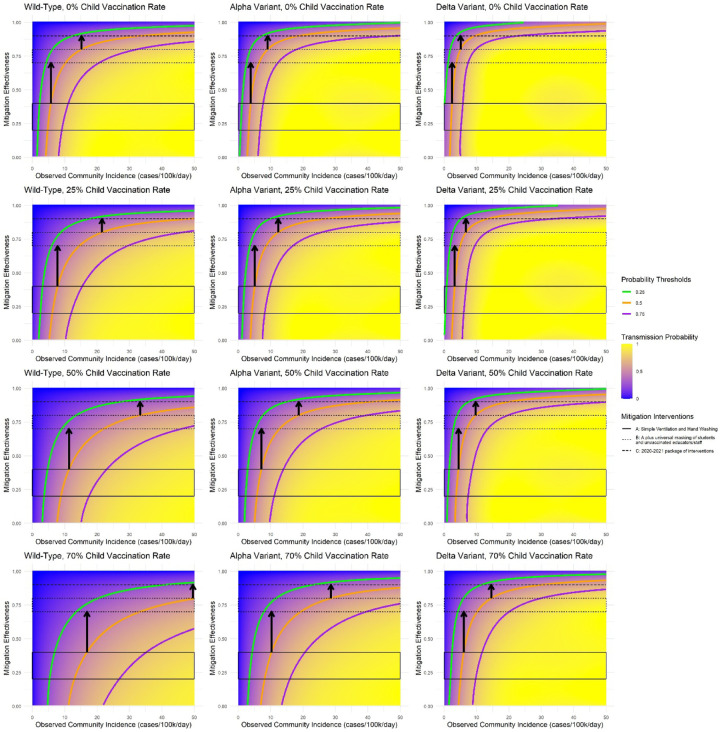
Model-estimated probability of at least one in-school SARS-CoV-2 transmission over 30 days in a simulated elementary school setting. The blue-to-yellow color scale depicts the smoothed model-estimated probability of at least one in-school SARS-CoV-2 transmission over a 30-day period. Panels reflect increasingly transmissible variants from left to right, and increasing student vaccination coverage from top to bottom. The horizontal axis shows observed community COVID-19 incidence in cases/100,000 people per day. The vertical axis shows mitigation effectiveness, applied as a relative risk reduction to the fully unmitigated attack rate for each variant. Bands of mitigation effectiveness reflect approximate assumptions for three types of interventions – see the Supplemental Table for more information about these effectiveness estimates. The contour lines represent thresholds for different probability levels; probabilities are lower than the threshold above the contour line and higher below it. The arrows indicate the community COVID-19 incidence rate at which a school might opt to move to the next more intensive mitigation strategy (i.e., 40% to 70% and 80% to 90% effectiveness), if the goal is to maintain the probability of the one in-school transmission per month below 50%. Adult vaccination coverage is assumed to be 70% in all scenarios (see Supplemental Figure 1 for a sensitivity analysis with 50% adult vaccination coverage).

**Figure 2:**
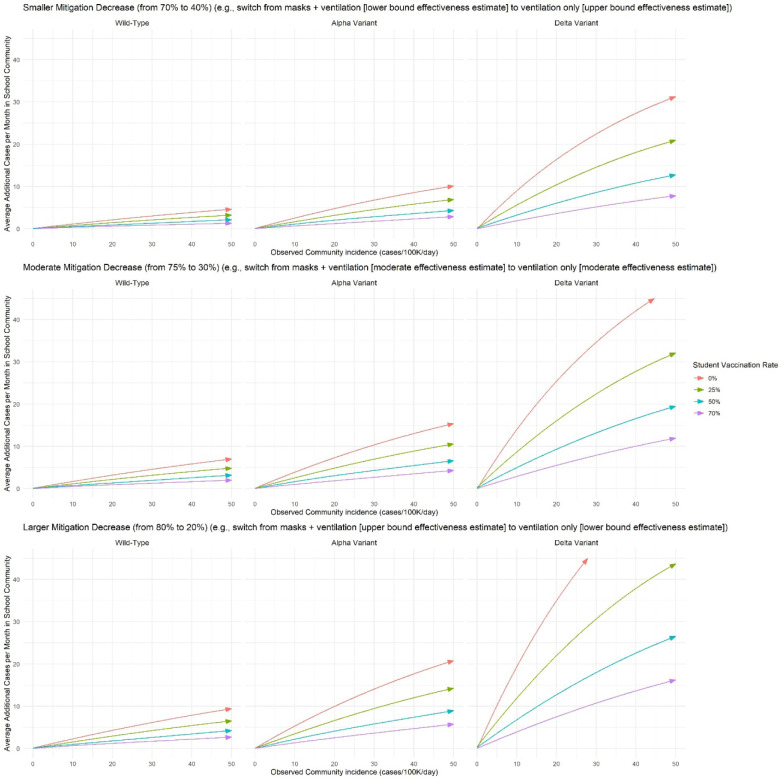
Model-estimated average number of additional cases over 30 days in the immediate school community (students, educators/staff, and their household members) associated with moving from intensive to less-intensive mitigation measures in the simulated elementary school setting. The vertical axis shows the smoothed average additional number of model-estimated infections over 30 days among members of the immediate school community (defined as students, educators/staff, and their household members) when moving from intensive to less intensive mitigation measures. The horizontal axis shows observed community COVID-19 incidence in cases/100,000 people per day. Panels reflect increasingly transmissible variants from left to right, and smaller differences in effectiveness between intensive and less intensive mitigation measures from top to bottom. The changes in mitigation effectiveness reflect the midpoints or bounds of the A and B mitigation scenarios presented in Figure 1: 70% to 40% mitigation effectiveness (smaller effectiveness decrease); 75% to 35% effectiveness (moderate effectiveness decrease); and 80% to 20% effectiveness (larger effectiveness decrease). Adult vaccination coverage is assumed to be 70% in all scenarios (see Supplemental Figure 2 for a sensitivity analysis with 50% adult vaccination coverage).

Over 30 days in the simulated elementary school, the probability of at least one in-school SARS-CoV-2 transmission varied widely (Figure 1). With the delta variant and no student vaccination, if decision-makers seek to keep the monthly probability of an in-school transmission below 50%, additional mitigation (e.g., masking) would need to be added to ventilation/handwashing at a community incidence of approximately 2/100,000/day, assuming 40% effectiveness of ventilation/handwashing (A) (Figure 1, top right panel, left arrow). As an alternative decision threshold, if decision-makers are willing to accept, in association with unmasking, an average of 5 additional infections per month in the immediate school community, masks could be removed at a community incidence of approximately 5/100,000/day, assuming 40% effectiveness of (A) and 70% effectiveness of (B) (Figure 2, top right panel, red line).

Increasing student vaccination to 50% in these two scenarios increased the associated thresholds to about 4 and 16 cases/100,000/day, respectively. Decreases in the assumed effectiveness of ventilation/handwashing, increases in the assumed effectiveness of masking, or decreases in the assumed proportion of all community infections that are detected shifted the thresholds lower.

## DISCUSSION

Despite high adult vaccination, the risks of in-school SARS-CoV-2 transmission and resulting infections among students, educators/staff, and their household members remain high when the delta variant predominates and students are unvaccinated. Mitigation measures or vaccinations for students can substantially reduce these risks, especially when implemented together.

Risks related to SARS-CoV-2 infection are only one of many factors guiding K-12 school planning, alongside educational, health, and social/emotional considerations. These results should be interpreted in the context of model limitations, including uncertainty in available data (e.g., effectiveness of individual mitigation interventions, case detection rate) and the focus on only the immediate school community; we excluded testing as a mitigation intervention (evaluated in previous work^3^). These findings underscore the potential role for responsive plans, where mitigation is deployed based on local COVID-19 incidence and vaccine uptake.

## Supplementary Material

1
